# Dietary L-Arginine and Zinc Oxide Nanoparticles Improve Growth Performance, Oxidative Status, Immunity, and Intestinal Integrity Indicators in Heat-Stressed Weaned Rabbits

**DOI:** 10.3390/vetsci13060598

**Published:** 2026-06-19

**Authors:** Tahani M. I. Al-Hazani, Amirah S. Alahmari, Manal A. Babaker, Ahmed M. Elbaz, Hagar E. Mohammed, Hany A. Thabet, Eman Kamel M. Khalfallah, Ahmed Ateya, Rowa K. Zarah, Khairiah Mubarak Alwutayd, Assem Abdou

**Affiliations:** 1 Biology Department, College of Science and Humanities, Prince Sattam bin Abdulaziz University, P.O. Box 83, Al-Kharj 11940, Saudi Arabia; t.alhazani@psau.edu.sa; 2 Department of Biology, College of Science, Princess Nourah bint Abdulrahman University, P.O. Box 84428, Riyadh 11671, Saudi Arabia; amsalahmari@pnu.edu.sa (A.S.A.); kmalwateed@pnu.edu.sa (K.M.A.); 3 Department of Chemistry, Faculty of Science, Majmaah University, Al Majmaah 11952, Saudi Arabia; m.babaker@mu.edu.sa; 4 Animal and Poultry Nutrition Department, Desert Research Center, Mataria, Cairo 11753, Egypt; dm.a.baz@gmail.com; 5 Zoology Department, Faculty of Science, Arish University, Al-Arish 45511, North Sinai, Egypt; hagarelmetwaly2017@gmail.com; 6 Poultry Production Department, Faculty of Agriculture, Ain Shams University, Cairo 11566, Egypt; hanyalieg@yahoo.com; 7 Biochemistry, Toxicology and Feed Deficiency Department, Animal Health Research Institute, Agricultural Research Center, Dokki, Giza 12616, Giza, Egypt; dr.emankamel@sci.asu.edu.eg; 8 Department of Development of Animal Wealth, Faculty of Veterinary Medicine, Mansoura University, Mansoura 35516, Dakahlia, Egypt; 9 Department of Biology, College of Science, Taif University, Taif 21944, Saudi Arabia; rowa.z@tu.edu.sa; 10 Animal Production Department, Faculty of Agriculture, Ain Shams University, Cairo 11566, Egypt; assemabdou@agr.asu.edu.eg

**Keywords:** rabbit performance, arginine, zinc nanoparticles, gut health, immunity, redox status, heat stress

## Abstract

This study evaluated the effects of adding zinc oxide nanoparticles (ZnNP), L-arginine (L-Arg), or a combination of both to the diets of growing rabbits to mitigate the physiological and productive consequences of heat stress. Two hundred and eighty 35-day-old New Zealand White rabbits were randomly assigned to four experimental treatments, with 70 rabbits per treatment and seven replicates (10 rabbits/replicate). The control group (Ctr) received the base diet without additives, while the diets of the other groups were fortified with arginine (L-Arg; 3 g/kg), zinc oxide nanoparticles (ZnNP; 40 mg/kg), or a combination of both (Arg-Zn). The results showed that the combined Arg-Zn significantly improved weight gain rate, feed conversion rate, carcass weight, and nutrient digestibility compared to the control group (*p* < 0.05). At the physiological level, we observed increased serum levels of total antioxidant capacity (T-AOC), glutathione peroxidase (GPx), superoxide dismutase (SOD), immunoglobulin G (IgG), immunoglobulin A (IgA), and triiodothyronine (T3), along with decreased levels of malondialdehyde (MDA), alanine aminotransferase (ALT), and aspartate aminotransferase (AST, *p* < 0.05) in Arg-Zn-fed rabbits. However, adding the Arg-Zn mixture contributed to a reduction in pathogenic bacteria counts and increased the volatile fatty acid (VFA) levels. At the molecular level, the gene expression of the inflammatory cytokines IL-6 and tumor necrosis factor alpha (TNF-α) decreased; however, the gene expression of claudins-1 (CLDN-1), cationic amino acid transporter-1 (CAT-1), mucin-2 (MUC-2), sodium-glucose co-transporter-1 (SGLT-1), and interferon gamma (IFNγ) increased (*p* < 0.05) in Arg-Zn-fed rabbits. These results suggest that dietary supplementation with ZnNP and L-Arg may serve as an effective nutritional strategy for improving growth performance, antioxidant status, immune function, and intestinal integrity in rabbits exposed to high ambient temperatures.

## Simple Summary

Heat stress is a major environmental challenge to sustainable rabbit production, particularly in hot and subtropical regions. It induces physiological, metabolic, and immune disturbances that impair digestive and immune functions, promote oxidative stress and systemic inflammation, and consequently reduce rabbit health, productivity, and ultimately lead to significant economic losses. In recent years, functional nutritional interventions have received considerable attention as a promising strategy for mitigating the adverse effects of heat stress in livestock. Among these interventions, L-arginine (L-Arg) has been reported to enhance nutrient utilization, support immune function, and modulate gut microbiota composition, whereas zinc oxide nanoparticles (ZnNP) possess antioxidant and anti-inflammatory properties that may help alleviate oxidative and physiological stress. Therefore, the present study was designed to evaluate the effects of combined dietary supplementation with L-Arg and ZnNP on heat-stressed growing rabbits. Specifically, the study investigated their effects on growth performance, antioxidant status, gut microbial populations, immune responses, and the expression of genes associated with intestinal health and immune regulation. We hypothesized that the combined supplementation would improve the ability of rabbits to cope with heat stress and thereby enhance their productive performance and overall health status.

## 1. Introduction

Rabbit meat is a valuable source of high-quality animal protein for human consumption [1]. It is characterized by its low fat and cholesterol contents, high protein content, and favorable amino acid profile, making it an important component of a healthy diet [2]. Moreover, rabbits exhibit high reproductive efficiency, rapid growth rates, and efficient feed conversion [3]. These characteristics make rabbit production a practical approach to enhancing food security and meeting the growing demand for animal protein, particularly in developing countries.

However, rabbit welfare and productivity are highly susceptible to environmental stressors, especially heat stress [3,4]. Rabbits have a limited capacity for heat dissipation due to their dense fur coat and the absence of functional sweat glands, making them particularly vulnerable to elevated ambient temperatures [5]. Exposure to heat stress can result in a range of physiological and metabolic disturbances, including reduced feed intake, impaired growth performance, decreased reproductive efficiency, and increased mortality [6]. These adverse effects are mediated, in part, through the induction of oxidative stress, which disrupts the balance between reactive oxygen species production and antioxidant defenses, thereby compromising immune function and cellular integrity [5,7]. In addition, heat stress can impair intestinal morphology and alter the composition of the gut microbiota, leading to reduced digestive efficiency, impaired nutrient absorption, and increased susceptibility to disease [8,9]. Collectively, these effects result in substantial economic losses and represent a major challenge to the sustainability of rabbit production systems. Therefore, developing effective nutritional and management strategies to alleviate the detrimental effects of heat stress is essential for maintaining rabbit health, welfare, and productivity.

Trace minerals are essential in rabbit nutrition, particularly zinc (Zn), which plays important roles in immunity, growth, metabolism, and antioxidant defense [10,11,12]. As a component of numerous enzymes, Zn supports protein and nucleic acid synthesis, cell division, tissue regeneration, and nutrient metabolism [13,14]. Zinc oxide nanoparticles (ZnNP) offer enhanced bioavailability due to their larger surface area and have been reported to possess antibacterial, immunomodulatory, and gut-health-promoting properties [14,15,16,17]. These benefits may be of great importance during heat stress, as increased production of reactive oxygen species (ROS) promotes oxidative damage, while ZnNP may mitigate these effects by enhancing antioxidant defenses [13,18,19]. Studies have reported improvements in growth performance, feed utilization, antioxidant status, immune response, reproductive traits, and intestinal health in rabbits supplemented with Zn or ZnNP [7,13,15,18].

Amino acids are essential nutrients that support protein synthesis, tissue repair, and various metabolic functions, thereby contributing to animal growth and performance [20,21]. Although rabbits can utilize microbial protein synthesized in the cecum through coprophagy, dietary amino acids remain necessary to meet the demands of growth, reproduction, and lactation [21,22]. Consequently, an adequate supply of essential amino acids is important for maintaining feed efficiency, health, and productivity [21]. Among these amino acids, arginine (L-Arg) plays a key role in protein synthesis, cell proliferation, and intestinal development, supporting muscle growth and overall performance [23,24]. As a precursor of nitric oxide, L-Arg also contributes to vasodilation and blood flow regulation, which may be particularly beneficial under heat stress conditions [25,26]. In addition, L-Arg supports immune function [27,28]. Therefore, adequate dietary L-Arg is important for maintaining immune competence, gut health, reproductive performance, and growth, especially during periods of stress [22,29]. Dietary L-arginine supplementation has been associated with enhanced growth performance, protein synthesis, antioxidant capacity, immune function, intestinal development, and reproductive performance in animals [22,27,29].

However, most previous studies have evaluated ZnNP or L-arginine separately, while information regarding their combined supplementation remains limited. Furthermore, the potential complementary effect between ZnNP and L-arginine on growth performance, physiological responses, antioxidant status, immune function, and related molecular mechanisms in rabbits has not been fully elucidated. Subsequently, it is assumed that adding them to the diets of heat-stressed rabbits may enhance their performance and health by supporting physiological function and intestinal integrity. Therefore, the present experiment was designed to address this knowledge gap by investigating the individual and combined effects of ZnNP and L-arginine supplementation, providing new insights into their potential complementary benefits and contributing to the development of more effective nutritional strategies for weaned rabbit production.

## 2. Materials and Methods

### 2.1. Experimental Additives

Zinc oxide nanoparticles (ZnNP) were obtained from the Nanoparticle Preparation Laboratory, National Research Centre, Egypt. The concentration of zinc oxide nanoparticles was 20 wt.% in distilled water, and their size was less than 40 nm. Dynamic light scattering (DLS) analysis confirmed a hydrodynamic diameter of <100 nm. The dispersion exhibited a pH of 7.5 ± 1.0 and a density of 1.7 ± 0.1 g/mL at 25 °C. The structural morphology, aggregation state, and structural features of the particle were studied through scanning electron microscopy (SEM) (JEOL Ltd., Akishima, Tokyo, Japan) and transmission electron microscopy (TEM) (JEOL Ltd., Akishima, Tokyo, Japan). Furthermore, the nanoparticles exhibited a spherical/irregular shape, and these analyses collectively confirm that the tested material falls within the nano-scale and displays properties consistent with engineered zinc oxide nanoparticles used in feed applications. ZnNPs were incorporated into the experimental diets by thoroughly premixing the required amount with a small quantity of the basal diet, followed by gradual mixing with the remaining feed to achieve a homogeneous distribution. Scanning electron microscopy was used to determine the structural morphology of the nanoparticles. High-purity L-arginine (98.5%) was purchased from a company that produces the Fufeng Group, and the company’s headquarters are located in Platinum Plaza, Laoshan District, Qingdao, 266114, China.

### 2.2. Animals and Diets

The experimental rabbits (New Zealand White, NZW) used in the rabbit breeding farm at the South Sinai station of the Desert Research Center were under the full joint supervision of the Faculty of Agriculture of Ain Shams University. Two hundred and eighty newly weaned rabbits, aged 35 days with an average weight of 768.6 ± 3.2 g, were randomly assigned to four experimental groups, each containing seven replicates (10 rabbits per replicate). The rabbits in the first group were fed the basic diet (Ctr), the rabbits in the second group were fed the basic diet with the addition of 3 g/kg arginine (L-Arg), the rabbits in the third group were fed the basic diet with the addition of 40 mg/kg zinc oxide nanoparticles (ZnNP), and the rabbits in the fourth group were fed the basic diet with the addition of L-Arg and ZnNP (Arg-Zn). The experiment lasted 42 days. Nutritional requirements were provided according to the breed requirements of the rabbits, in accordance with the recommendations of the National Research Council [30], as shown in Table 1. Water and feed were provided continuously throughout the trial period. After preparing the experimental feed, it was stored in dry, airtight containers at room temperature.

### 2.3. Rearing Conditions

The experiment was conducted in June 2025 during the summer season at the Desert Research Center, Egypt. The rabbits were housed in an open-sided, well-ventilated building equipped with exhaust fans to ensure continuous air exchange through side vents, thereby reducing heat accumulation inside the facility. Animals were kept in individual cages under standard management conditions, with ad libitum access to feed and clean drinking water throughout the experimental period. Each rabbit was housed in an individual cage with dimensions of approximately 60 × 50 × 40 cm (length × width × height), allowing adequate space for normal movement and minimizing stress during the experimental period. Temperature and relative humidity were recorded daily between 1:00 and 2:00 p.m., and the average values during the experimental period were 30.8 ± 2.1 °C and 56.3 ± 4.8%, respectively. The Temperature–Humidity Index (THI) was calculated to evaluate heat stress conditions, as described by Alqhtani et al. [4]. The THI values ranged from 29.1 to 31.3, indicating that the rabbits were exposed to heat stress conditions as previously defined by Elbaz et al. [6]. The lighting program consisted of 16 h light and 8 h dark. To prevent interference with the dietary treatments, no antibiotics or therapeutic medications were administered during the experiment. Strict hygiene practices were implemented, including routine cleaning and removal of fecal waste to maintain sanitary conditions. The animals were monitored daily by a qualified veterinarian to ensure health and welfare throughout the study.

### 2.4. Performance Measurements

Live body weight and feed intake were measured twice during the experimental period at 56 and 77 days of age, and then daily weight gain (DWG, g), daily feed intake (DFI, g) and feed conversion ratio (FCR, g/g) were calculated to assess growth performance. Twenty-eight rabbits (7 from each group) were slaughtered at 77 days of age to evaluate carcass characteristics. Carcass weight and internal organs (heart, kidneys, lungs, spleen, liver, and abdominal fat) were weighed as described by Alqhtani et al. [2]. All carcass traits were expressed as percentages relative to live body weight at slaughter using the formula (organ or carcass weight/live body weight) × 100, to standardize comparisons among animals of different body weights. Furthermore, after the experimental period, 7 rabbits from each group were isolated in individual digesters to evaluate nutrient digestibility, including dry matter, crude protein, ether extract, crude fiber, and nitrogen-free extract. After placing the digestive cages individually, feces were collected twice daily for four days. The feed intake was dried after the quantity was recorded, and the feces were dried to assess the target digestibility coefficients based on AOAC [31].

### 2.5. Blood Parameters

At the end of the experimental period, seven rabbits from each treatment group were randomly selected and slaughtered according to standard procedures. During the slaughter process, blood samples were collected and processed for hematological and biochemical analyses, including the assessment of immune status, oxidative stability, lipid profile, thyroid hormones, and liver enzyme activities in serum. After drawing blood samples in tubes, centrifugation (4000× *g* at 10 min) was performed, and they were then stored at −20 °C until the necessary analyses could be carried out. Total protein, albumin, aspartate aminotransferase (AST), alkaline phosphatase (ALP), alanine aminotransferase (ALT), glucose, cholesterol, triglycerides, high-density lipoprotein (HDL), and low-density lipoprotein (LDL) levels were determined using commercial kits produced by Stanbio Laboratory (Boerne, TX, USA) and measured with a spectrophotometer (Shimadzu UV-1601) (Shimadzu Corporation, Kyoto, Japan). The activity of antioxidant enzymes was also assessed using a specialized commercial suite from Life Diagnostics that includes T-AOC (total antioxidant capacity), SOD (superoxide dismutase), GPx (glutathione peroxidase), and MDA (malondialdehyde) (West Chester, PA, USA). Levels of T3 (triiodothyronine) and T4 (thyroxine) hormones were measured to assess thyroid function, according to the steps of Ibrahim et al. [32]. Immunoglobulin G, M, and A (IgG, IgM, and IgA) were measured by ELISA kits according to the manufacturer’s instructions (Shanghai Liquid Quality Testing Technology Co., Ltd., Shanghai, China) and absorbance was measured with a BioTek ELx800 Microplate Reader (BioTek Instruments, Winooski, VT, USA).

### 2.6. Gut Measurements

At 77 days of age, during slaughter, pH and volatile fatty acid (VFA) concentrations were measured, and intestinal contents were collected for microbial count from seven rabbits per group. To measure the VFA concentration in the cecal contents, gas chromatography was employed to determine the concentrations of acetate, butyrate, and propionate. In addition, the pH of fresh cecal contents was determined using a pH meter (PHSJ-3F, Leici, Shanghai, China). The cecal contents collected during slaughter were immediately placed in a sterile bag for analysis and stored at −80 °C to evaluate selected microbial communities. Sequencing from 10^−1^ to 10^−8^ of the cecal contents was performed, and the samples were then placed in the appropriate agar medium for each microorganism at the specified temperature and time. In MRS agar, *Lactobacillus* was cultured at 37 °C for 37 for 72 h. In MacConkey agar, *E. coli* (*Escherichia coli*) was cultured at 35 °C for 35 for 24 h. In TSC agar, *C. perfringens* (*Clostridium perfringens*) was cultured at 35 °C for 37 for 48 h. In XLD agar, *Salmonella* was cultured at 37 °C for 48 h. To assess intestinal inflammatory factors, a portion of the duodenum was resected and cleaned with saline, and the mucosa scraped. The mucosa samples were stored at −80 °C until targeted analyses were performed. Additionally, using ELISA kits, secretory immunoglobulin A (sIgA), interleukin 1α (IL-1α), and interleukin 2 (IL-2) levels were assessed according to the manufacturer’s instructions (Shanghai Liquid Quality Testing Technology Co., Ltd., Shanghai, China), as described by Ye et al. [33].

### 2.7. mRNA Expression Analysis

Total RNA was extracted from jejunal mucosa using the AxyPrep Multisource Total RNA Miniprep Kit (Axygen Biosciences, Inc., Union City, CA, USA), following the manufacturer’s instructions with a modification that included DNase treatment between the two wash steps. Briefly, columns were incubated for 30 min at 37 °C with a mixture containing RNase-free DNase (1 U/μL), reaction buffer, and DEPC-treated water. RNA quantity and integrity were assessed using the Qubit Fluorometer (Thermo Fisher Scientific, Waltham, MA, USA) and Agilent 2100 Bioanalyzer (Agilent Technologies, Santa Clara, CA, USA), respectively.

The purity and concentration of extracted RNA were assessed spectrophotometrically using the A260/A280 ratio, and only samples exhibiting acceptable purity (1.8–2.0) were used for subsequent analyses. Amplification specificity was verified by melt-curve analysis, which demonstrated a single peak for each primer pair, confirming the absence of nonspecific amplification and primer–dimer formation. Primer efficiencies were evaluated using standard curve analysis and were within the acceptable range for quantitative PCR. Each experimental group included seven biological replicates, and all reactions were performed in triplicate. No-template controls (NTC) and no-reverse-transcription controls (No-RT) were included in each run to exclude contamination and genomic DNA amplification. The expression stability of *GAPDH* was evaluated across all dietary treatments and heat-stress conditions and showed no significant variation among groups; therefore, GAPDH was selected as the endogenous reference gene for normalization of target gene expression.

First-strand cDNA was synthesized from 5 μg total RNA using the SuperScript III First-Strand Synthesis SuperMix (Thermo Fisher Scientific, Waltham, MA, USA). The resulting cDNA was purified, quantified using a NanoDrop ND-1000 (Thermo Fisher Scientific, Waltham, MA, USA), and stored at −20 °C until use. Gene expression analysis was performed by RT-qPCR using SYBR Green chemistry on a GeneAmp 7900 (Applied Biosystems, Foster City, CA, USA). Primers targeting the following genes were designed as shown in Table 2: *MUC-2*: mucin-2; *CLDN-1*: claudins-1; *CAT-1*: cationic amino acid transporter-1; *SGLT-1*: sodium–glucose co-transporter-1; *IL-6*: interleukin 6; *TNF-α*: tumor necrosis factor alpha; *IFNγ:* interferon gamma; and the reference gene (*GAPDH*). Relative gene expression was calculated according to the method (2^−∆∆Ct^ method) described by Livak and Schmittgen [34] with the GAPDH gene used as control group.

### 2.8. Data Statistical Analysis

To assess the effects of arginine and nano-zinc supplementation on heat stress, the general linear model method was used in SPSS version 18.0 (SPSS Inc., Chicago, IL, USA). One-way analysis of variance (ANOVA) and Tukey’s multiple comparisons test were used to compare differences between all groups. The homogeneity and normality of the experimental data were assessed by Shapiro–Wilk tests. Each animal was considered the experimental unit for all experimental measures except for growth measures, where the experimental unit was the replicate. Statistical significance was set at *p* < 0.05. Data are presented in tables and figure as means and ±SD.

## 3. Results

### 3.1. Growth Indicators

The effect of arginine, zinc oxide nanoparticles, or their combination on growth management is shown in Table 3. During the 35–56-day period, DWG (*p* < 0.01) and DFI (*p* < 0.05) were higher in rabbits fed L-Arg, ZnNP, and Arg-Zn compared to Ctr-fed rabbits. However, FCR was lower in rabbits fed L-Arg, ZnNP, and Arg-Zn compared to Ctr-fed rabbits (*p* < 0.01). During the 57–77-day period, DWG was higher in rabbits fed Arg-Zn compared to rabbits fed L-Arg, ZnNP, and control; however, DFI was unaffected among the experimental groups (*p* ≥ 0.05). Moreover, FCR was lower in rabbits fed L-Arg, ZnNP, and Arg-Zn (*p* < 0.01) compared to Ctr-fed rabbits. During the 35–77-day period, DWG was higher in Arg-Zn-fed rabbits compared to rabbits fed L-Arg, ZnNP, and Ctr (*p* < 0.01); however, DFI was unaffected among the experimental groups (*p* ≥ 0.05). Additionally, FCR was lower in rabbits fed L-Arg, ZnNP, and Arg-Zn compared to Ctr-fed rabbits (*p* < 0.01).

### 3.2. Carcass Traits

The effect of arginine, zinc oxide nanoparticles, or their combination on carcass traits is shown in Table 4. Carcass weight increased in rabbits fed L-Arg and Arg-Zn compared to rabbits fed ZnNP and Ctr (*p* = 0.003), with the highest carcass weight observed in the Arg-Zn group. Additionally, abdominal fat decreased in rabbits fed L-Arg, ZnNP, and Arg-Zn compared to Ctr-fed rabbits, with the lowest abdominal fat content observed in the Arg-Zn and L-Arg groups (*p* < 0.01). Despite that, the remaining carcass characteristics, including of the heart, kidney, spleen, lungs, and liver, were not affected by the experimental treatments (*p* ≥ 0.05).

### 3.3. Nutrient Digestibility

Table 5 shows the effect on nutrient digestibility of adding arginine, zinc oxide nanoparticles, or their combination in heat-stressed rabbits. Although the digestibility of crude fiber, ether extract, and nitrogen-FE was unaffected (*p* ≥ 0.05) by the experimental additives, the digestibility of crude protein increased in rabbits fed L-Arg, ZnNP, and Arg-Zn compared to Ctr-fed rabbits (*p* < 0.01). Furthermore, the digestibility of dry matter was increased in rabbits fed L-Arg and Arg-Zn compared to rabbits fed ZnNP and Ctr (*p* = 0.015).

### 3.4. Serum Metabolites

Table 6 shows the effect on serum metabolites of adding arginine, zinc oxide nanoparticles, or their combination in heat-stressed rabbits. Although total protein levels were higher in rabbits fed L-Arg and Arg-Zn compared to ZnNP- and Ctr-fed rabbits (*p* = 0.011), albumin levels were not affected (*p* ≥ 0.05) by the experimental supplements. Additionally, glucose, cholesterol, triglycerides, and LDL levels were lower in L-Arg, ZnNP, and Arg-Zn rabbits compared to Ctr-fed rabbits (*p* < 0.01). HDL levels were higher in L-Arg, ZnNP, and Arg-Zn rabbits compared to Ctr-fed rabbits (*p* < 0.01).

### 3.5. Vital Functions

Table 7 shows the effect on thyroid and liver function and oxidative status of adding arginine, zinc oxide nanoparticles, or a combination thereof in rabbits subjected to heat stress. Regarding thyroid activity, T3 levels increased in rabbits supplemented with L-Arg, ZnNP, and Arg-Zn compared to Ctr-fed rabbits (*p* < 0.01); however, T4 levels were unaffected (*p* ≥ 0.05). Additionally, liver enzyme levels decreased, with ALT (*p* = 0.012) and AST (*p* = 0.008) levels lowering in rabbits supplemented with L-Arg, ZnNP, and Arg-Zn compared to Ctr-fed rabbits; however, ALP levels were unaffected (*p* ≥ 0.05). Regarding oxidation enzymes, the activity of SOD, T-AOC, and GPx enzymes increased (*p* < 0.01) in the groups supplemented with L-Arg, ZnNP, and Arg-Zn compared to the Ctr group. Moreover, T-AOC (*p* = 0.015) and SOD (*p* < 0.01) levels were highest in the groups receiving Arg-Zn and ZnNP. Similarly, serum MDA content decreased in the groups supplemented with L-Arg, ZnNP, and Arg-Zn compared to the Ctr group (*p* < 0.01); however, it was lowest in the Arg-Zn group.

### 3.6. Immunity

Table 8 illustrates the role of arginine supplementation, zinc oxide nanoparticles, or a combination in modifying immunity in heat-stressed rabbits. IgA, IL-2, and sIgA levels increased in the rabbits that received L-Arg, ZnNP, and Arg-Zn supplements (*p* < 0.05) compared to the control group. Similarly, IgG levels tended to increase in the rabbits that received ZnNP and Arg-Zn supplements compared to the L-Arg and Ctr groups (*p* = 0.020); however, IgM and IL-1α levels were unaffected (*p* ≥ 0.05).

### 3.7. Gut Health

Supplementation with arginine or zinc oxide nanoparticles thereof demonstrated an antimicrobial effect, as shown in Table 9. *E. coli* and *C. perfringens* populations decreased (*p* < 0.01 and *p* = 0.011, respectively) in the groups receiving L-Arg, ZnNP, and Arg-Zn compared to the Ctr group (*p* < 0.05); however, the *Salmonella* population was unaffected (*p* ≥ 0.05). Additionally, the *Lactobacillus* population increased (*p* < 0.01) in the groups receiving L-Arg, ZnNP, and Arg-Zn compared to the Ctr group, while the highest population count was observed in the Arg-Zn and L-Arg groups (*p* < 0.01). Additionally, butyrate concentration increased in the rabbits that received L-Arg, ZnNP, and Arg-Zn compared to the Ctr (*p* < 0.01). However, the concentration of acetate in the rabbits that received L-Arg, ZnNP, and Arg-Zn tended to increase (*p* = 0.027) compared to Ctr. Nevertheless, the concentration of propionate and the pH value did not change between the experimental groups (*p* ≥ 0.05). However, the pH value in the intestines of the rabbits that received L-Arg and Arg-Zn decreased numerically compared to the Ctr and ZnNP (*p* = 0.061).

### 3.8. Gene Expression Assessment

**Figure 1:**
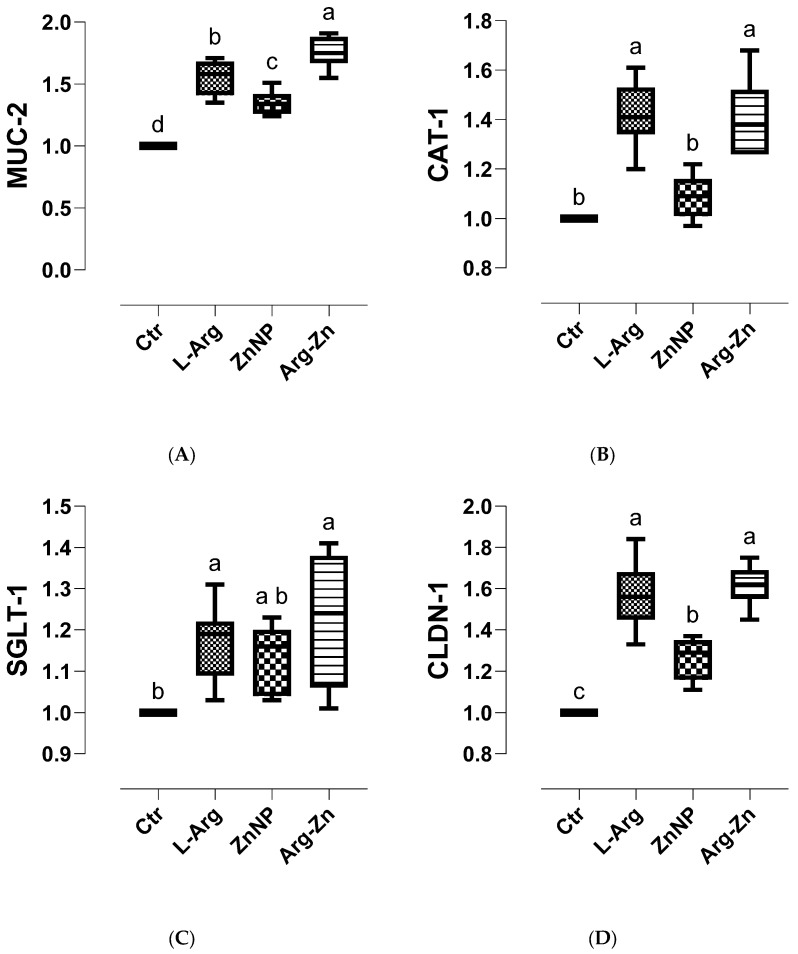
Effect of supplementation with L-arginine, zinc oxide nanoparticles, and their combination on mucin-2 (MUC-2, (**A**)), cationic amino acid transporter-1 (CAT-1, (**B**)), sodium–glucose co-transporter-1 (SGLT-1, (**C**)), and claudins-1 (CLDN-1, (**D**)) mRNA expression in heat-stressed weaned rabbits. Ctr: group-fed basal diet without supplements; L-Arg: group-fed diet including arginine supplements; ZnNP: group-fed diet including zinc oxide nanoparticle supplements; Arg-Zn: group-fed diet including arginine and zinc oxide nanoparticles. Different lowercase letters (a–d) above the bars indicate significant differences among groups (*p* < 0.05).

**Figure 2:**
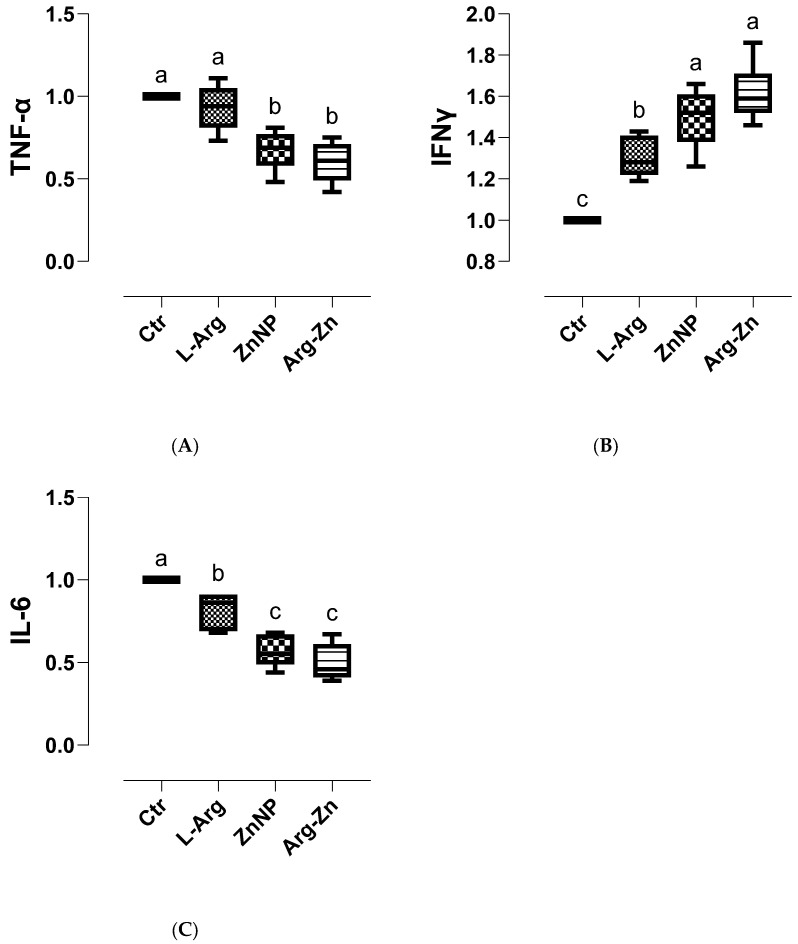
Effect of supplementation with L-arginine, zinc oxide nanoparticles, and their combination on tumor necrosis factor alpha (TNF-α, (**A**)), interferon gamma (IFNγ, (**B**)), and interleukin-6 (IL-6, (**C**)) mRNA expression in heat-stressed weaned rabbits. Ctr: group-fed basal diet without supplements; L-Arg: group-fed diet including arginine supplements; ZnNP: group-fed diet including zinc oxide nanoparticle supplements; Arg-Zn: group-fed diet including arginine and zinc oxide nanoparticles. Different lowercase letters (a–c) above the bars indicate significant differences among groups (*p* < 0.05).

Figure 1 and Figure 2 illustrate the effect of arginine or zinc oxide nanoparticle supplements on gene expression related to intestinal health and nutrient digestion and absorption. Adding L-Arg and Arg-Zn increased the expression of the SGLT-1, CLDN-1, and CAT-1 genes (*p* < 0.05) compared to the other groups (Figure 1). Furthermore, adding L-Arg, ZnNP, and Arg-Zn increased the expression of the MUC-2 and IFNγ genes compared to the Ctr group (*p* < 0.01). However, IL-6 gene expression reduced in rabbits receiving L-Arg and ZnNP, and Arg-Zn, while TNF-α gene expression lowered in rabbits receiving ZnNP and Arg-Zn (*p* < 0.01) compared to the other groups (Figure 2).

## 4. Discussion

Heat stress remains a major obstacle to rabbit production due to its detrimental effects on performance and health, as supported by the results of this study. Therefore, studying the effects of both arginine and zinc oxide nanoparticles on the health of weaned rabbits, especially during heat stress, has significant productive implications. We hypothesize that the Arg-Zn mixture may support immunity, intestinal integrity, antioxidant capacity, and overall health in weaned rabbits, thus mitigating the significant effects associated with heat stress and weaning, thereby promoting performance. To verify our hypothesis, this study was conducted, which showed that L-Arg and ZnNP supplementation had a positive effect on the performance of heat-stressed rabbits by improving nutrient digestion, blood lipid levels, and antioxidant status, as well as modifying immunity and gene expression associated with gut health.

In this study, supplementation with the Arg-Zn mixture improved the growth performance of heat-stressed weaned rabbits, as evidenced by increased body weight and improved feed conversion ratio. The enhanced growth response may be attributed to the complementary physiological roles of arginine and zinc in regulating nutrient utilization, immune function, antioxidant defense, and metabolic activity [22,35]. Arginine serves as a precursor for nitric oxide synthesis, which promotes blood flow, nutrient delivery, and cellular metabolism, whereas nano-zinc acts as an essential cofactor for numerous enzymes involved in protein synthesis, antioxidant protection, and immune regulation [22,35]. The beneficial effects of the Arg-Zn mixture may also be associated with improvements in intestinal health and function. Zinc contributes to the maintenance of epithelial integrity and digestive efficiency through its antioxidant properties, thereby supporting nutrient absorption [36,37]. Concurrently, L-Arg enhances intestinal development by increasing villus height, reducing intestinal permeability, and modulating inflammatory responses, resulting in improved gut functionality and nutrient utilization [22,27,28,38,39]. In the present study, these effects were further supported by the upregulation of genes involved in nutrient transport and intestinal barrier function, including SGLT-1, CAT-1, MUC-2, and CLDN-1. Additionally, both nano-zinc and L-Arg have demonstrated antimicrobial and microbiota-modulating properties [10,16,23,27]. Their combined supplementation may promote the proliferation of beneficial microorganisms and increase short-chain fatty acid production, thereby enhancing energy harvest and protein utilization [40]. Furthermore, the results of the current study indicate a significant increase in the expression of IFN-γ and a decrease in the expression of IL-6 and TNF-α, which suggests improved immune system efficiency and reduced inflammatory stress. Therefore, by supporting intestinal health, reducing inflammation, and strengthening antioxidant defenses, the Arg-Zn mixture may enable a greater proportion of dietary nutrients and metabolic energy to be directed toward growth rather than stress adaptation. These findings are consistent with previous reports showing that dietary supplementation with either nano-zinc or L-Arg improves growth performance and feed efficiency in rabbits [14,18,22]. Therefore, the improved growth performance observed in rabbits receiving the Arg-Zn mixture likely reflects the integrated effects of enhanced nutrient transport, intestinal barrier function, antioxidant protection, and immune regulation, as well as modulating intestinal microbial populations.

Consistent with the growth results, the incorporation of the Arg-Zn mixture increased carcass weight and resulted in a significant decrease in abdominal fat in the current study. The increase in carcass weight is likely due to the effect of arginine and zinc in supporting intestinal health, which enhances nutrient digestion and absorption, thus increasing nutrient availability [7,12,39]. Zinc supplements play vital roles in protein metabolism and cell growth, and are involved in the activity of many digestive enzymes [35]. They also contribute to antioxidant defense, maintain the integrity of the intestinal barrier, and promote villus growth [10,41], thereby enhancing muscle mass and increasing carcass weight. Furthermore, L-Arg stimulates the secretion of growth hormones to regulate protein and amino acid metabolism [40], thereby improving nucleic acid stability, promoting protein precipitation in cells, and regulating cell proliferation [27,28], and thus improving carcass weight. Additionally, L-Arg enhances nutrient absorption for muscle growth through the growth of intestinal villi, blood flow to intestinal tissues, and mucosal integrity, thus supporting tissue growth and metabolic processes [26,29]. Furthermore, the addition of the Arg-Zn mixture reduces abdominal fat in heat-stressed rabbits, according to this study. This reduction in abdominal fat may be attributed to the effect of arginine and zinc supplementation in improving fat metabolism, promoting protein synthesis and nitrogen retention [42,43,44], and enhancing the efficient allocation of nutrients to muscle tissue growth. Therefore, the Arg-Zn mixture can improve carcass characteristics by enhancing nutrient digestion and absorption, which is consistent with several previous reports [45,46].

According to the results of the current study, adding the Arg-Zn mixture enhanced the digestibility of dry matter and crude protein. This improvement is attributed to the properties of both L-Arg and zinc. Previous reports have demonstrated zinc’s antibacterial properties, as well as its role in the synthesis of several enzymes that regulate protein and nucleic acid metabolism, thus modulating metabolic rate [47,48]. Furthermore, intestinal health is closely linked to efficient digestion and nutrient absorption in animals. This is where L-Arg plays a role, contributing to intestinal recovery during heat stress by supporting intestinal morphology and function, and by regenerating the intestinal epithelium [39,42,45], thereby enhancing nutrient digestibility. This supports our study’s findings that adding the Arg-Zn mixture may support digestive function, thereby improving nutrient digestibility and absorption.

Blood biochemical indices are valuable indicators of the physiological and metabolic status of rabbits exposed to heat stress and can be used to evaluate the efficacy of nutritional interventions in maintaining metabolic homeostasis and liver function [8,37]. In the present study, supplementation with the Arg–Zn mixture increased serum total protein and HDL concentrations while reducing glucose, total cholesterol, triglycerides, and LDL concentrations. The increase in total protein may be associated with the role of L-Arg in nitrogen metabolism, protein synthesis, and protein turnover regulation [22,49]. Improved nitrogen utilization and enhanced anabolic processes may have contributed to the higher circulating protein concentration observed in the supplemented groups. In addition, L-Arg has been reported to influence lipid metabolism through the modulation of enzymes involved in fatty acid synthesis and utilization [22]. Nitric oxide, a major metabolite of arginine, may also participate in the regulation of lipid metabolic pathways, including those related to lipogenesis, thereby contributing to improvements in the blood lipid profile [50]. Zinc may have further contributed to these effects through its involvement in numerous enzymatic processes associated with lipid metabolism, energy utilization, and cellular homeostasis [51,52,53]. Moreover, the antioxidant properties of zinc help maintain hepatocellular integrity and support normal liver function, which may favorably influence lipid metabolism under heat-stress conditions [12,52]. The reductions in serum cholesterol, triglycerides, and LDL concentrations, together with the increase in HDL concentration, suggest an improvement in lipid metabolism and metabolic balance in rabbits receiving the Arg–Zn mixture. These findings are consistent with previous studies reporting beneficial effects of dietary nano-zinc and L-Arg supplementation on serum protein concentration and lipid profile in rabbits [12,22,37].

Additionally, the present results demonstrated that supplementation with the Arg–Zn mixture improved antioxidant status and was associated with favorable changes in liver- and thyroid-related indicators in heat-stressed rabbits. Specifically, T-AOC, GPx, SOD, and T3 concentrations increased, whereas ALT, AST, and MDA concentrations decreased following Arg–Zn supplementation. These effects may be partly attributed to the complementary antioxidant properties of arginine and zinc. L-Arg serves as the substrate for nitric oxide synthase, which catalyzes nitric oxide production. Nitric oxide improves vascular function by promoting vasodilation and enhancing blood flow, thereby facilitating oxygen and nutrient delivery to tissues [54,55]. L-Arg has also been reported to support glutathione synthesis, an important component of the cellular antioxidant defense system that helps protect cell membranes from oxidative damage, particularly under heat-stress conditions [56,57]. In addition, nitric oxide may contribute to the regulation of cellular redox balance and antioxidant enzyme activity through mechanisms that have been associated with Nrf2-mediated antioxidant pathways [22,58], thereby reducing lipid peroxidation and lowering MDA concentrations. Zinc may further enhance antioxidant defenses through its involvement in the structure and activity of several antioxidant enzymes, particularly superoxide dismutase [12]. Moreover, zinc has been shown to stimulate metallothionein synthesis and may reduce reactive oxygen species generation by limiting metal-catalyzed oxidative reactions, thereby helping to protect cellular components from oxidative damage [10,58,59,60,61]. These actions may contribute to membrane stability and the maintenance of normal intestinal barrier function under heat stress. The improved antioxidant status observed in rabbits receiving the Arg–Zn mixture may have contributed to the favorable changes in liver- and thyroid-related indicators. By supporting cellular redox homeostasis and reducing oxidative stress, arginine and zinc may help preserve hepatic integrity, as reflected by the lower ALT and AST concentrations, and support thyroid hormone homeostasis, which may partly explain the higher circulating T3 concentrations [3,23,48,57,62]. Overall, these findings are consistent with previous studies reporting beneficial effects of arginine and zinc supplementation on antioxidant status and organ function in rabbits exposed to environmental stressors [12,23,37]. The observed improvements suggest a potential role of the Arg–Zn mixture in alleviating oxidative stress and supporting metabolic homeostasis during heat stress.

Heat stress impairs immune function in rabbits by suppressing both humoral and cellular immune responses, compromising intestinal barrier integrity, and promoting inflammatory processes [21,63]. In the present study, dietary supplementation with the Arg–Zn mixture increased serum IgA, IgG, sIgA, and IL-2 concentrations, suggesting an enhancement of selected immune-related indicators in heat-stressed rabbits. The observed increase in immunoglobulin concentrations may be associated with the immunomodulatory properties of both L-Arg and zinc. Immunoglobulin G plays an important role in systemic immune defense, whereas IgA and sIgA contribute to mucosal immunity by limiting pathogen adherence to mucosal surfaces and supporting barrier protection [22,37,64,65]. L-Arg has been reported to influence immune responses through its involvement in nitric oxide synthesis and its regulatory effects on lymphocyte function and antibody production [22,65]. In addition, L-Arg may support the development and activity of B cells, thereby enhancing humoral immune responses. Additionally, zinc is an essential trace element for immune cell proliferation, differentiation, and function. Adequate zinc availability supports B-cell activity and antibody production, which may contribute to the increased immunoglobulin concentrations observed in the present study [18,37]. Zinc also plays a critical role in maintaining intestinal mucosal integrity, thereby supporting local immune defenses and sIgA production [13,19,37]. Furthermore, zinc is required for the growth, maturation, and activation of T lymphocytes, which may contribute to the elevated IL-2 concentrations observed in supplemented rabbits [66,67,68]. Previous studies have also suggested that L-Arg may influence gut microbial activity and the production of microbial metabolites with immunoregulatory properties, which could indirectly support immune function [69]. The present findings are consistent with earlier reports demonstrating beneficial effects of L-Arg and zinc supplementation on immune-related parameters in rabbits [22,37,70,71]. These results suggest that the Arg–Zn mixture may help alleviate heat-stress-associated immune suppression and support immune homeostasis in growing rabbits.

Heat stress disrupts intestinal microbial counts, leading to alterations in cecal fermentation and reduced production of beneficial microbial metabolites such as short-chain fatty acids (SCFAs). These changes may impair epithelial integrity, compromise intestinal immune function, and increase susceptibility to intestinal inflammation [3,6,8]. Therefore, nutritional interventions that support intestinal homeostasis may help alleviate the adverse effects of heat stress. In the present study, supplementation with the Arg–Zn mixture was associated with favorable changes in the cecal microbial ecosystem, as evidenced by reduced counts of *E. coli* and *C. perfringens*, increased *Lactobacillus* populations, and higher concentrations of acetate and butyrate. These findings suggest an improvement in cecal microbial counts and fermentation activity under heat-stress conditions. Previous studies have demonstrated a close relationship between zinc availability and gut microbial ecology [72]. Zinc may influence microbial composition by supporting intestinal health and creating conditions that favor the proliferation of beneficial bacteria while limiting the growth of opportunistic pathogens [73,74]. The increased abundance of *Lactobacillus* observed in the present study may have contributed to the suppression of pathogenic microorganisms through the production of antimicrobial metabolites and fermentation products, including volatile fatty acids [74]. Consistent with these findings, previous reports have shown that zinc supplementation can increase populations of beneficial bacteria, including *Lactobacillus* and *Bacillus subtilis* [74,75,76]. Similarly, dietary L-Arg supplementation has been reported to influence gut microbial composition and reduce pathogen colonization in animals challenged with *Salmonella typhimurium* or *C. perfringens* [27,28]. The combined effects of L-Arg and nano-zinc may therefore contribute to the establishment of a more favorable intestinal microbial environment. Overall, the present findings suggest that the Arg–Zn mixture may help modulate the gut microbiome and support cecal fermentation during heat stress, thereby contributing to improved intestinal health and physiological stability in growing rabbits.

Volatile fatty acids (VFAs), primarily acetate, propionate, and butyrate, are the principal end products of microbial fermentation of complex carbohydrates in the rabbit cecum [77]. These metabolites play important roles in gastrointestinal physiology and energy metabolism, with butyrate serving as a major energy source for intestinal epithelial cells [77,78]. The increased VFA concentrations observed in the present study may be associated with the combined effects of L-Arg and zinc on cecal fermentation processes. Zinc has been reported to support carbohydrate metabolism and may indirectly influence microbial activity and fermentation efficiency within the gastrointestinal tract [78,79]. Similarly, previous studies have suggested that dietary L-Arg supplementation can alter cecal microbial populations and metabolic activity, potentially affecting the production of VFAs and other fermentation-derived metabolites [27,28,80]. VFAs contribute to the maintenance of intestinal health through several mechanisms, including supporting epithelial cell metabolism, promoting mucin production, enhancing intestinal barrier function, and helping maintain a favorable luminal environment that may limit the proliferation of pathogenic microorganisms [80,81,82,83]. Previous reports have also shown that beneficial bacterial populations can produce VFAs and antimicrobial compounds, such as bacteriocins, which may further support gut health and microbial balance [84]. Collectively, the increased VFA concentrations observed in rabbits receiving the Arg–Zn mixture suggest a potential improvement in cecal fermentation activity and intestinal health under heat-stress conditions. However, because microbial composition was not directly assessed in the present study, the specific mechanisms underlying these changes warrant further investigation.

Gene expression analysis provides valuable molecular insights into the physiological responses of rabbits exposed to heat stress [85]. Heat stress can alter the expression of genes involved in nutrient transport, intestinal barrier function, oxidative balance, and immune regulation, thereby affecting animal health and productivity [8]. Previous studies have suggested that nutritional interventions may modulate these molecular responses and help maintain physiological homeostasis under stressful environmental conditions [6,35,86]. In the present study, dietary supplementation with the Arg–Zn mixture upregulated the expression of SGLT-1, CAT-1, MUC-2, CLDN-1, and IFN-γ, while downregulating IL-6 and TNF-α expression. The increased expression of SGLT-1 and CAT-1 suggests an enhanced capacity for intestinal nutrient transport. SGLT-1 encodes a sodium-dependent glucose transporter located on the apical membrane of intestinal epithelial cells and plays an important role in glucose and monosaccharide absorption [85]. Similarly, CAT-1 encodes a transporter responsible for the uptake and transport of cationic amino acids, including arginine, across cellular membranes [6]. The upregulation of MUC-2 and CLDN-1 indicates potential improvements in intestinal barrier function. MUC-2 encodes mucin-2, the principal structural component of the intestinal mucus layer, which protects epithelial cells from physical and microbial challenges [4,86,87]. CLDN-1 encodes claudin-1, a key tight-junction protein involved in maintaining epithelial integrity and regulating paracellular permeability [88]. The enhanced expression of these genes may be associated with the favorable changes observed in cecal microbial populations and fermentation products following Arg–Zn supplementation. Previous studies have reported that beneficial microbial metabolites, particularly short-chain fatty acids, may influence epithelial function and the expression of genes associated with nutrient transport and barrier integrity [80,81]. Therefore, the increased concentrations of acetate and butyrate observed in the present study may have contributed to the upregulation of SGLT-1, CAT-1, MUC-2, and CLDN-1 expression. In addition, the Arg–Zn mixture modulated the expression of immune-related genes, as evidenced by increased IFN-γ expression and reduced expression of the pro-inflammatory cytokines IL-6 and TNF-α. These findings suggest a shift toward a more balanced immune response and reduced intestinal inflammatory signaling under heat-stress conditions. IFN-γ plays an important role in immune regulation and host defense by activating immune cells and enhancing antimicrobial responses [9,27,89,90]. Overall, these results suggest that the Arg–Zn mixture may support nutrient transport, intestinal barrier integrity, and immune homeostasis through modulation of gene expression, thereby contributing to improved intestinal health and physiological resilience during heat stress. A limitation of the present study is the absence of a thermoneutral control group; therefore, future studies should include both thermoneutral and heat-stressed rabbits to better evaluate the efficacy of these dietary supplements under different environmental conditions.

## 5. Conclusions

Our results indicate that dietary supplementation with a combination of L-arginine and zinc oxide nanoparticles improved growth performance and several physiological indicators in rabbits exposed to high ambient temperatures. The Arg–Zn mixture enhanced antioxidant status, as evidenced by increased selected antioxidant enzyme activities and immunoglobulin levels, along with reduced malondialdehyde concentrations. The Arg–Zn mixture supplementation was also associated with favorable changes in selected stress-related indicators, including thyroid activity, as well as reductions in liver enzyme activities and glucose levels. In addition, the Arg–Zn mixture supplementation was associated with favorable changes in selected cecal microbial populations and increased volatile fatty acid concentrations. Moreover, it modulated the expression of genes related to nutrient absorption, inflammation, and intestinal tight junction integrity, which may suggest a potential improvement in intestinal functionality under heat-stress conditions. Under the conditions of this study, dietary inclusion of the Arg–Zn combination may contribute to improved physiological responses and intestinal health indicators in heat-stressed rabbits. Further studies, including thermoneutral control groups and more detailed mechanistic investigations, are recommended to confirm these effects.

## Figures and Tables

**Table 1:** Chemical evaluation and composition of fed basal diets.

Ingredients	%
Alfalfa hay powder	40.0
Soybean meal	13.0
Corn	12.1
Wheat bran	15.8
Barley	13.5
Sunflower meal	2.00
Molasses	2.00
Salt	0.30
Dicalcium phosphate	0.50
Limestone	0.50
Premix *	0.30
**Nutrient level**	
Energy (MJ/kg)	10.08
Crude protein	17.52
Crude fiber	14.23
Total phosphorus	0.49
Calcium	0.86

Premix * = Premix contains the following per kg: Vitamin A: 12,000 IU; Vitamin E: 50 mg; Vitamin D3: 1000 IU; Iron: 50 mg; Manganese: 30 mg; Selenium: 0.08 mg; Copper: 5 mg; Zinc: 50 mg; Iodine: 0.1 mg; and Cobalt: 0.2 mg.

**Table 2:** Primers used for real-time PCR.

Target Genes	GenBank ID	Primer Sequences—Sense	Primer Sequences—Antisense
*MUC-2*	L41544.1	TATACCGCAAGCAGCCAGGT	GCAAGCAGGACACAGACCAG
*CAT-1*	XM_002721425.3	CCAGTCTATTAGGTTCCATGTTCC	CGATTATTGGCGTTTTGGTC
*SGLT-1*	NM_001101692.1	GATTTCCCGTATGATTACCGAG	AAGAGGGAGACAACCACAACG
*CLDN1*	NM_001089316.1	GGAGCAAAAGATGCGGATGG	AATTGACAGGGGTCAAAGGGT
*TNF-α*	XM_008262537.2	CTACGTGGGCTAGAGGCTTG	TTCTTCAGCCTCACTCTCTCC
*IL-6*	NC_013678	GCCAACCCTACAACAAGA	AGAGCCACAACGACTGAC
*IFNγ*	NM_001081991.1	TTCTTCAGCCTCACTCTCTCC	TGTTGTCACTCTCCTCTTTCC
*GAPDH*	NC_013676.1	TGTTTGTGATGGGCGTGAA	CCTCCACAATGCCGAAGT

**Table 3:** Effect of supplementation with L-arginine, zinc oxide nanoparticles, and their combination on growth performance in heat-stressed weaned rabbits.

	Parameter	Ctr	L-Arg	ZnNP	Arg-Zn	*p* Value
35–56 days	IBW (g)	769.2 ± 8.43	767.8 ± 7.17	768.3 ± 8.03	769.1 ± 6.64	0.872
	DWG (g)	22.4 ± 1.93 ^c^	25.1 ± 1.85 ^b^	24.5 ± 2.08 ^b^	26.4 ± 1.91 ^a^	<0.001
	DFI (g)	45.8 ± 2.51 ^b^	49.2 ± 3.01 ^a^	48.9± 2.99 ^a^	49.5 ± 2.84 ^a^	0.016
	FCR (g/g)	2.04 ± 0.09 ^a^	1.96 ± 0.05 ^b^	1.99 ± 0.07 ^b^	1.87 ± 0.08 ^c^	0.001
57–77 days	DWG (g)	28.2 ± 3.52 ^b^	28.5 ± 2.93 ^b^	28.0 ± 3.22 ^b^	29.0 ± 2.77 ^a^	0.003
	DFI (g)	164 ± 5.26	158 ± 4.65	158 ± 4.38	156 ± 5.04	0.058
	FCR (g/g)	5.84 ± 0.10 ^a^	5.54 ± 0.08 ^c^	5.66 ± 0.06 ^b^	5.36 ± 0.07 ^d^	<0.001
35–77 days	DWG (g)	24.6 ± 2.73 ^c^	26.1 ± 2.05 ^ab^	25.5 ± 2.48 ^b^	27.1 ± 2.34 ^a^	<0.001
	DFI (g)	101 ± 4.52	99.8 ± 3.81	99.9 ± 4.03	98.8 ± 3.92	0.513
	FCR (g/g)	4.11 ± 0.15 ^a^	3.82 ± 0.09 ^b^	3.90 ± 0.11 ^b^	3.66 ± 0.10 ^c^	<0.001

Ctr: group-fed basal diet without supplements; L-Arg: group-fed diet including arginine supplements; ZnNP: group-fed diet including zinc oxide nanoparticle supplements; Arg-Zn: group-fed diet including arginine and zinc oxide nanoparticles. IBW: initial body weight; DWG: daily weight gain; DFI: daily feed intake; FCR: feed conversion ratio. ^a−d^ Means within the same row with different superscripts differ significantly (*p* < 0.05).

**Table 4:** Effect of supplementation with L-arginine, zinc oxide nanoparticles, and their combination on carcass characteristics in heat-stressed weaned rabbits.

Parameter	Ctr	L-Arg	ZnNP	Arg-Zn	*p* Value
Pre-slaughter (g)	1814 ± 5.81 ^c^	1871 ± 4.15 ^b^	1853 ± 5.22 ^b^	1906 ± 4.37 ^a^	<0.001
CW (%)	69.6 ± 3.32 ^c^	71.8 ± 2.81 ^b^	70.5 ± 3.16 ^c^	72.7 ± 2.62 ^a^	0.003
Heart (%)	0.29 ± 0.08	0.30 ± 0.06	0.28 ± 0.03	0.30 ± 0.09	0.255
Kidney (%)	0.71 ± 0.13	0.69 ± 0.09	0.70 ± 0.06	0.71 ± 0.11	0.318
Lungs (%)	0.67 ± 0.05	0.66 ± 0.03	0.68 ± 0.07	0.67 ± 0.04	0.107
Liver (%)	3.12 ± 0.16	3.23 ± 0.25	3.15 ± 0.12	3.21 ± 0.17	0.436
Spleen (%)	0.051 ± 0.07	0.052 ± 0.03	0.054 ± 0.05	0.056 ± 0.04	0.071
A. Fat (%)	2.74 ± 0.35 ^a^	2.08 ± 0.44 ^c^	2.31 ± 0.29 ^b^	2.04 ± 0.31 ^c^	0.001

Ctr: group-fed basal diet without supplements; L-Arg: group-fed diet including arginine supplements; ZnNP: group-fed diet including zinc oxide nanoparticle supplements; Arg-Zn: group-fed diet including arginine and zinc oxide nanoparticles. A. Fat: abdominal fat; CW: carcass weight. ^a–c^ Means with no common superscript in the same row are significantly different (*p* < 0.05).

**Table 5:** Effect of supplementation with L-arginine, zinc oxide nanoparticles, and their combination on nutrient digestibility in heat-stressed weaned rabbits.

Parameter	Ctr	L-Arg	ZnNP	Arg-Zn	*p* Value
Dry matter (%)	65.7 ± 5.8 ^b^	67.3 ± 3.7 ^a^	66.2 ± 4.9 ^b^	67.8 ± 4.5 ^a^	0.015
Crude fiber (%)	51.3 ± 2.4	51.7 ± 3.1	50.8 ± 2.1	51.6 ± 2.6	0.194
Crude protein (%)	67.0 ± 1.8 ^c^	69.4 ± 1.5 ^a^	68.3 ± 1.7 ^b^	69.7 ± 1.4 ^a^	0.001
Ether extract (%)	80.1 ± 3.5	79.6 ± 4.6	80.4 ± 3.8	81.0 ± 3.3	0.082
NFE (%)	49.7 ± 5.1	50.5 ± 3.9	50.1 ± 4.7	50.8 ± 4.2	0.447

Ctr: group-fed basal diet without supplements; L-Arg: group-fed diet including arginine supplements; ZnNP: group-fed diet including zinc oxide nanoparticle supplements; Arg-Zn: group-fed diet including arginine and zinc oxide nanoparticles. NFE: nitrogen-free extract. ^a–c^ Values within rows followed by different superscript letters differ significantly (*p* < 0.05).

**Table 6:** Effect of supplementation with L-arginine, zinc oxide nanoparticles, and their combination on serum metabolites in heat-stressed weaned rabbits.

Parameter	Ctr	L-Arg	ZnNP	Arg-Zn	*p* Value
Total protein (g/dL)	5.03 ± 1.7 ^b^	5.26 ± 1.9 ^a^	5.09 ± 1.3 ^b^	5.32 ± 1.4 ^a^	0.011
Albumin (g/dL)	3.28 ± 1.2	3.31 ± 0.7	3.26 ± 1.1	3.37 ± 0.9	0.319
Glucose (mg/dL)	153 ± 2.8 ^a^	128± 2.4 ^c^	141 ± 3.2 ^b^	126 ± 2.1 ^c^	<0.001
Cholesterol (mg/dL)	214 ± 5.0 ^a^	186 ± 4.3 ^b^	193 ± 4.8 ^ab^	181 ± 3.9 ^b^	0.001
Triglycerides (mg/dL)	173 ± 3.5 ^a^	159 ± 2.9 ^b^	170 ± 3.8 ^a^	161 ± 3.1 ^b^	0.006
HDL (mg/dL)	40.7 ± 1.3 ^c^	42.6 ± 0.9 ^b^	44.2 ± 0.5 ^a^	44.8 ± 0.8 ^a^	0.001
LDL (mg/dL)	86.3 ± 1.8 ^a^	83.5 ± 1.5 ^b^	81.7 ± 2.1 ^c^	81.2 ± 1.7 ^c^	<0.001

Ctr: group-fed basal diet without supplements; L-Arg: group-fed diet including arginine supplements; ZnNP: group-fed diet including zinc oxide nanoparticle supplements; Arg-Zn: group-fed diet including arginine and zinc oxide nanoparticles. LDL: low-density lipoprotein; HDL: high-density lipoprotein. ^a–c^ Values within rows followed by different superscript letters differ significantly (*p* < 0.05).

**Table 7:** Effect of supplementation with L-arginine, zinc oxide nanoparticles, and their combination on thyroid hormones, liver enzymes, and antioxidant enzymes in heat-stressed weaned rabbits.

Parameter		Ctr	L-Arg	ZnNP	Arg-Zn	*p* Value
Thyroid hormones	T 3 (ng/dL)	2.11 ± 0.50 ^c^	2.28 ± 0.71 ^b^	2.34 ± 0.60 ^ab^	2.42 ± 0.42 ^a^	0.001
	T 4 (ng/dL)	117.0 ± 2.40	115.2 ± 2.15	119.3 ± 2.71	118.0 ± 2.37	0.091
Liver enzymes	ALT (U/L)	19.21 ± 1.61 ^a^	16.40 ± 1.30 ^b^	17.63 ± 1.13 ^b^	16.23 ± 1.41 ^b^	0.012
	AST (U/L)	63.50 ± 2.00 ^a^	59.32 ± 1.33 ^b^	60.71 ± 1.50 ^b^	58.82 ± 1.73 ^b^	0.008
	ALP (U/L)	164.03 ± 0.82	161.10 ± 0.52	159.21 ± 0.91	160.20 ± 0.82	0.103
Antioxidant enzymes	MDA (nmol/mL)	2.73 ± 0.21 ^a^	1.94 ± 0.42 ^b^	1.25 ± 0.33 ^c^	0.87 ± 0.65 ^d^	<0.001
	T-AOC (U/mL)	1.48 ± 0.63 ^b^	1.53 ± 0.16 ^b^	1.94 ± 0.16 ^a^	2.03 ± 0.21 ^a^	0.015
	GPx (U/mL)	24.60 ± 1.70 ^c^	25.63 ± 1.93 ^b^	26.71 ± 1.30 ^b^	27.43 ± 1.57 ^a^	0.001
	SOD (U/mL)	37.21 ± 0.41 ^c^	39.50 ± 0.14 ^b^	42.36 ± 0.52 ^a^	43.12 ± 0.36 ^a^	<0.001

Ctr: group-fed basal diet without supplements; L-Arg: group-fed diet including arginine supplements; ZnNP: group-fed diet including zinc oxide nanoparticle supplements; Arg-Zn: group-fed diet including arginine and zinc oxide nanoparticles. ALP: albumin; ALT: alanine aminotransferase; AST: aspartate aminotransferase; T 3: triiodothyronine; T 4: thyroxine; MDA: malondialdehyde; T-AOC: total antioxidant capacity; SOD: superoxide dismutase; GPx: glutathione peroxidase. ^a−d^ Means within rows with different superscripts differ significantly (*p* < 0.05).

**Table 8:** Effect of supplementation with L-arginine, zinc oxide nanoparticles, and their combination on immunity in heat-stressed weaned rabbits.

Parameter		Ctr	L-Arg	ZnNP	Arg-Zn	*p* Value
Serum	IgA (ug/mL)	207 ± 0.94 ^c^	233 ± 0.51 ^b^	246 ± 0.73 ^a^	252 ± 0.61 ^a^	0.001
	IgM (ug/mL)	391 ± 3.05	402 ± 2.81	396 ± 3.28	411 ± 2.15	0.117
	IgG (ug/mL)	12.6 ± 1.48 ^b^	13.1 ± 1.08 ^ab^	13.8 ± 1.55 ^a^	14.2 ± 1.37 ^a^	0.020
Duodenum	IL-2 (ug/g)	43.1 ± 1.24 ^c^	47.6 ± 2.05 ^b^	46.8 ± 1.64 ^b^	50.3 ± 1.95 ^a^	<0.001
	IL-1α (ug/g)	16.7 ± 0.30	16.3 ± 0.15	17.1 ±0.27	17.5 ± 0.34	0.097
	sIgA (ug/g)	9.4 ± 0.08 ^b^	11.7 ± 0.11 ^a^	11.3 ± 0.05 ^a^	12.2 ± 0.09 ^a^	0.003

Ctr: group-fed basal diet without supplements; L-Arg: group-fed diet including arginine supplements; ZnNP: group-fed diet including zinc oxide nanoparticle supplements; Arg-Zn: group-fed diet including arginine and zinc oxide nanoparticles. sIgA: secretory immunoglobulin A; IL-1α: interleukin 1α; IL-2: interleukin 2; immunoglobulin G, M, and A (IgG, IgM, and IgA). ^a–c^ Values within rows followed by different superscript letters differ significantly (*p* < 0.05).

**Table 9:** Effect of supplementation with L-arginine, zinc oxide nanoparticles, and their combination on gut health in heat-stressed weaned rabbits.

Parameter		Ctr	L-Arg	ZnNP	Arg-Zn	*p* Value
VFA concentration	Propionate	1.75 ± 0.12	1.81 ± 0.09	1.73 ± 0.05	1.80 ± 0.08	0.143
	Butyrate	6.13 ± 0.26 ^c^	6.54 ± 0.31 ^a^	6.27 ± 0.15 ^b^	6.61 ± 0.19 ^a^	0.001
	Acetate	28.4 ± 0.70 ^b^	29.7 ± 0.62 ^a^	29.1± 0.54 ^ab^	30.2 ± 0.63 ^a^	0.027
	pH	6.47 ± 0.33	6.36 ± 0.41	6.44 ± 0.35	6.32 ± 0.49	0.061
Microbial enumeration (log10 CFU/g digesta)	*E. coli*	5.81 ± 0.23 ^a^	4.42 ± 0.35 ^b^	4.62 ± 0.28 ^b^	4.53 ± 0.24 ^b^	<0.001
	*C. perfringens*	4.35 ± 0.09 ^a^	4.03 ± 0.11 ^b^	4.11 ± 0.06 ^b^	4.06 ± 0.07 ^b^	0.011
	*Salmonella*	1.92 ± 0.02	1.88 ± 0.05	1.94 ± 0.08	1.84 ± 0.04	0.217
	*Lactobacillus*	6.08 ± 0.37 ^c^	7.15 ± 0.21 ^a^	6.48 ± 0.34 ^b^	7.27 ± 0.26 ^a^	<0.001

Ctr: group-fed basal diet without supplements; L-Arg: group-fed diet including arginine supplements; ZnNP: group-fed diet including zinc oxide nanoparticle supplements; Arg-Zn: group-fed diet including arginine and zinc oxide nanoparticles. *E. coli*: *Escherichia coli*; *C. perfringens*: *Clostridium perfringens*. ^a−c^ Means within rows with different superscripts differ significantly (*p* < 0.05).

## Data Availability

The original contributions presented in this study are included in the article. Further inquiries can be directed to the corresponding authors.

## Abbreviations

The following abbreviations are used in this manuscript:

A. fat	Abdominal fat
ALT	Alanine aminotransferase
Arg-Zn	Group-fed diet including arginine and zinc oxide nanoparticles
AST	Aspartate aminotransferase
*C. perfringens*	*Clostridium perfringens*
CAT-1	Cationic amino acid transporter-1
CLDN-1	Claudin-1
Ctr	Group-fed basal diet without supplements
CW	Carcass weight
DFI	Daily Feed intake
DWG	Daily weight gain
*E. coli*	*Escherichia coli*
GPx	Glutathione peroxidase
FCR	Feed conversion ratio
HDL	High-density lipoprotein
IFN-γ	Interferon-gamma
IL-1β	Interleu-kin-1β
IL-6	Interleukin 6
L-Arg	Group-fed diet including arginine supplements
LDL	Low-density lipoprotein
MUC-2	Mucin-2
NFE	Nitrogen-free extract
SOD	Superoxide dismutase
T-AOC	Total antioxidant capacity
TNF-α	Tumor necrosis factor-alpha
ZnNP	Group-fed basal diet including zinc oxide nanoparticles
